# A PhotoVoice Study with Canadian Immigrant and Racialized Family Caregivers of Children on the Autism Spectrum

**DOI:** 10.3390/ijerph23020222

**Published:** 2026-02-10

**Authors:** Jesse Sam, Farah Ahmad, Tareq Khalaf, Anjana Sathies, Sukaina Dada

**Affiliations:** 1 School of Health Policy and Management, York University, Toronto, ON M3J 1P3, Canada; dr.jessesam@gmail.com (J.S.); tareqk@yorku.ca (T.K.);; 2 SMILE Canada—Support Services, Toronto, ON M1T 3V3, Canada; sukaina.dada@smilecan.org

**Keywords:** autism, family caregiver, immigrant, racialized, photovoice

## Abstract

**Highlights:**

**Public health relevance: How does this work relate to a public health issue?**

- Given the lifelong nature of autism and the complexity of needed care, a public health approach is needed that centers on the families living with autism.
- Yet, perspectives of caregiving families are overlooked, especially those from marginalized communities.

**Public health significance: Why is this work of significance to public health?**

- Unmet needs of family caregivers lead to stress, burnout, and mental health strain, which have population-level effects on family health and service consumption.
- This study fills a key evidence gap by centering on immigrant and racialized caregivers’ voices, a group that remains underrepresented in autism and caregiving research despite facing disproportionate structural disadvantage.

**Public health implications: What are the key implications or messages for practitioners, policy makers, and/or researchers in public health?**

- By documenting family caregivers’ lived experiences using a participatory PhotoVoice approach, this study highlights how social determinants of health, including racism, language barriers, immigration status, and service fragmentation, directly affect access to autism care and family health.
- The findings show the need for culturally responsive, coordinated health and education systems that support family caregivers across transitions, reduce administrative burden, and address stigma and exclusion within schools and autism-related services.

**Abstract:**

Background: Immigrant and racialized families raising children on the autism spectrum in Canada navigate intersecting inequities shaped by racism, language barriers, immigration status, and fragmented health and education systems. Yet their perspectives remain underrepresented in autism and health policy research. Methods: Guided by the socioecological and critical social science lens, this community-based participatory study employed a PhotoVoice approach in partnership with SMILE Canada–Support Services. Ten immigrant and/or racialized family caregivers from the Greater Toronto area participated in four in-person sessions involving ethical training, guided photo-taking, group-based reflections, and collaborative theme refinement. The data included 38 participant-generated photographs, narratives, and an audio-recorded final group discussion. Results: Seven interrelated themes were identified: (1) family support and child needs; (2) physical and emotional burden on caregivers; (3) school support or its missingness; (4) stigma and discrimination; (5) overall journey with barriers; (6) transitions and uncertainty; and (7) two sides of a coin: isolation and strength, loneliness and hope. Caregivers highlighted extensive invisible labor, exclusionary schooling, financial and systemic barriers, and cumulative stress. Simultaneously, they articulated resilience, mutual support, and a strong sense of collective responsibility. The PhotoVoice process itself was experienced as validating, unifying, and empowering, with participants expressing readiness to disseminate findings through exhibitions, school boards, universities, and policy-engagement initiatives. Conclusions: Caregiving among immigrant and racialized families is both a profound act of love and a site of structural injustice. Centering on caregivers as co-researchers and knowledge holders reveals urgent needs for equity-oriented autism policies and culturally responsive, accessible support systems in Canada.

## 1. Introduction

Autism is a lifelong and complex neurodevelopmental condition that influences how people communicate, learn, and experience the world. Although the prevalence of autism in many low- and middle-income countries is not known, the World Health Organization estimates that, on average, 1 in 127 people are living with autism [1]. More recent systematic reviews suggest a higher prevalence in some regions, with global estimates closer to 1 in 100 [2]. The rates are high in developed countries due to growing awareness about autism and the availability of qualified professionals to make the diagnosis. In Canada, autism affects about 1 in 50 children and youths aged 1 to 17 years [3]. The support needs for people living with autism vary and range from mild to moderate or high, while many of them live with more than one diagnosis, such as developmental coordination disorder, attention-deficient hyperactivity disorder, anxiety, intellectual disability, sleep problems, gastrointestinal problems, and so forth [4]. As the diagnosis of autism typically occurs in early childhood years, parents become the primary caregivers, and other family members, like siblings, uncles, and aunts, sometimes join as the support needs continue and change over time. 

Family caregivers’ ongoing support plays a central role in the lives of children and youths on the autism spectrum [5]. Beyond everyday parenting, they manage intensive and evolving responsibilities, including daily care, supporting communication, coordinating appointments, and coping with stress and stigma [6,7,8]. Although Canadian scholars report the overall experiences of family caregivers of children on the autism spectrum, considerably less attention has been paid to the experiences of immigrant and racialized families [9]. In the Canadian context, the term *racialized* describes people or groups who are labeled or treated as belonging to a minority race, not because of biology, but because of social perceptions, and who often experience unequal treatment as a result [10]. A handful of studies on equity-deserving groups have started to document their compounded barriers that limit access to appropriate care and support [11,12,13,14]. This is concerning given Canada’s increasing population diversity since the 1960s, when the immigration policy moved from a preferential support of European applicants to a point-based system with priority settlement criteria (e.g., education, age, work experience, and language proficiency), opening doors to applicants from all countries. Recently, the census of 2021 showed that nearly one-quarter of Canadians are first-generation immigrants and that the majority of them are non-White [15]. Using the same census data, scholars also reported that among 11 racialized groups, 10 had a higher poverty rate than the White Canadian population [16]. These demographic shifts, socioeconomic inequities, and increase in rates of autism call attention to the urgency of examining and addressing the social and structural determinants of well-being among racialized and immigrant families raising children on the autism spectrum.

With the overarching aim of advancing scholarly insights and strengthening equity in policymaking and practice surrounding autism-related supports, we engaged with racialized and immigrant Canadian family caregivers of children and youths on the spectrum through PhotoVoice using a community-based participatory approach. The specific objectives of this study were twofold: first, to bring immigrant and racialized caregivers’ perspectives to the forefront of research and policy conversations, and, second, to empower participants as advocates within their communities. 

### Theoretical Underpinnings

Our research approach drew from the emancipatory critical social science perspective. We believe that reality is shaped by context and crystallizes over time as *historical realism*, and, thus, humans have the agency to transform the environment they live in and experience in intertwined ways [17,18]. This research orientation enhanced reflexivity and guided us to look beyond the medical model in understanding autism and caregiving. 

While autism has often been framed through a medical model with individual-level focus on diagnosis, treatment, and normalization [19], alternative perspectives such as the social model of disability [20] and the neurodiversity paradigm shift attention to the ways in which societal barriers, rather than individual deficits, primarily shape the lives of people with autism [21,22,23]. According to Najeeb and Quadt [24], we can determine that these perspectives are particularly relevant for critical public health research because they foreground the social, cultural, and structural factors that influence well-being. To operationalize our theoretical underpinning, we incorporated the socioecological framework [25,26]. The socioecological framework recognizes caregiving experiences as shaped through interactions across multiple, interconnected levels, including the individual and family (microsystem), relationships between families and institutions such as schools and health services (mesosystem), policies and structures (exosystem), and broader social norms and values (macrosystem). This framework functioned as an organizing analytic lens to situate the participants’ narratives within layered social environments and examine how barriers and supports emerged, interacted, and accumulated across systems.

Being reflexive researchers, we consider it important to share our positionality. We identify ourselves as immigrant and/or racialized researchers with an interdisciplinary perspective that draws from disciplines like public health, health policy and systems, critical disability studies, and medicine. We share a passion for supporting families living with autism or other disabilities and have been engaged with families in professional roles either directly or through the agencies serving them. These positionalities informed the research process in important ways regarding trust, openness to ask reflexive questions, attention to power relations, assumptions and interpretive decisions, and the use of suitable reflexive strategies, as discussed later.

## 2. Methods

To meet the study objectives, we selected a PhotoVoice participatory research method [27] and drew from the principles of community-based participatory research (CBPR), which emphasizes collaboration, co-learning, and shared ownership of knowledge production [28,29,30]. Prior to any data collection, this study received ethical approval from the Human Participants Review Sub-Committee at York University, Toronto, Canada.

In PhotoVoice participatory research, participants engage in photography and dialogue, enabling them to capture, reflect on, and share experiences and issues important to them [27]. This approach is especially helpful to engage with participants from marginalized populations as it facilitates raising their critical consciousness on the issues they select and the identification of pathways for social change [27,31]. Given that PhotoVoice combines participant-generated photography with collective reflection, individuals are likely to capture aspects of their lives that may be difficult to express in traditional interviews or surveys. The novelty of PhotoVoice lies in the use of the SHOWeD technique for collective analysis, whereby participants are invited to reflect on five guiding questions about each photograph they take: *What do you **S**ee? What is **H**appening? How does this relate to **O**ur lives? Why does this issue **e**xist? What can we **D**o about it?* [27]. The prompt *Why does this issue exist?* was expanded by asking the participants to reflect on *individual, family, community, culture, system, and societal levels.* We deemed the PhotoVoice approach as suitable to serve the dual objectives of our study: to gain equity-enhancing scholarly insights and achieve participant empowerment. To the best of our knowledge, the PhotoVoice approach has not been previously used to engage with Canadian immigrant and racialized family caregivers of children on the autism spectrum, and our work is anticipated to demonstrate its potential. 

As a first step in our study guided by the CBPR principles, we established *collaboration* with the community-based organization SMILE Canada—Support Services [SMILE hereafter], which serves racialized and immigrant family caregivers of people living with disabilities including autism in the Greater Toronto Area. SMILE follows a peer-navigator model and offers services in several languages, including English, Urdu, Arabic, Farsi, Dari, and Somali. The researchers and SMILE staff members, including those with lived experiences, had several meetings that facilitated the development of the study protocol. SMILE helped in several ways, such as with feedback on the protocol, distribution of the study flyer through its listserv, and the provision of space for the study sessions. The CBPR principle of *co-learning* was executed in several ways. For instance, we reflected on the ongoing debate within the autism community regarding person-first language (e.g., “children with autism”) versus identity-first language (e.g., “autistic children”) [32,33]. We invited our study participants to comment, and they preferred person-first language; thus, we opted to use person-first language for the rest of our work. The family caregivers in the study were not only participants but also *co-researchers* whose perspectives shaped the research questions, discussions, and interpretations. Likewise, the *ownership* principle was followed by making sure the ownership of photographs taken by the participants remained with them, and they freely engaged in sharing the photograph booklet with practitioners and policymakers. This alignment between PhotoVoice and CBPR was central to this study’s equity goals: to highlight the voices of immigrant and racialized family caregivers to advance scholarly insights and empower them in sharing gained insights for culturally responsive policy and practice. 

### 2.1. Settings and Participants

This study took place in the Greater Toronto Area (GTA), Canada’s largest metropolitan region, home to over 7 million residents and one of the most culturally diverse cities globally [34]. Data from the 2021 Census indicate that 46.6% of the population (2,862,850 individuals) are immigrants and 13.7% of them arrived between 2016 and 2021. In the 2021 census, the top three places of birth for immigrants in Toronto City were India, China, and the Philippines [34]. 

PhotoVoice participant eligibility criteria included adult family caregivers of children or youth with a formal autism diagnosis who self-identified as immigrants and/or racialized individuals residing in the GTA and were proficient in English. A purposive sampling strategy was employed to recruit family caregivers who could provide in-depth insights into navigating autism-related support, caregiving demands, and interactions with community and institutional systems as immigrant and/or racialized families. Additionally, snowball sampling was utilized to expand reach within the community, particularly among caregivers not connected to formal service systems, which led to the recruitment of two participants. Overall, 10 eligible family caregivers joined the PhotoVoice study, with ages ranging from early 20s to late 50s (Table 1). Seven participants were immigrants, primarily from East Africa, South Asia, and Eastern Europe. While most participants were mothers, extended family caregivers, including siblings and aunts, were also represented. Employment status varied, with five participants unemployed and others engaged in part-time or informal work. Self-reported social support ranged from poor to excellent, often influenced by extended family presence and service access. Because this study is part of a larger project, nine of the participants who joined the PhotoVoice study were also part of our previous focus group study.

### 2.2. Data Collection and Analysis

All participants received study information in detail and provided written informed consent prior to each of the four sessions. Four in-person PhotoVoice sessions were conducted, each lasting approximately two hours. Sessions were designed to build trust [35], reduce hierarchy, and foster collaborative dialogue [31]. Holding the sessions in a trusted community space reduced researcher–participant hierarchy and fostered openness. For each session, the seating was arranged in a circle, and culturally appropriate refreshments were provided. Additional safeguards included culturally sensitive communication and the availability of SMILE support staff in case of need. Many participants were already familiar with the project from earlier involvement, which further enhanced trust and comfort. Each participant received a $100 honorarium for each session.

Session 1 focused on introducing participants to the PhotoVoice methodology, discussing ethical considerations in photography, providing practical training on photo-taking techniques, explaining the SHOWeD method, and sharing the emergent findings of the recently completed focus groups with family caregivers on their experiences of accessing supports for their family members on the spectrum. Through discussion, the PhotoVoice study participants identified two key areas for exploration through PhotoVoice: Family and Community Support and the School and Education System. Next, participants were invited to take photographs over one week on their related experiences and write reflective notes using SHOWeD prompts to explain the meaning behind the images. Participants submitted their photographs to the research team via WhatsApp or email prior to Session 2. The taking of new photos and writing of reflective notes was repeated by participants between Sessions 2 and 3 over a two-week period. 

Sessions 2 and 3 focused on sharing and interpreting photographs. Participants first discussed their images and narratives in small groups and collaboratively named each photo. They then participated in a “walk-around” gallery exercise, where all photographs and stories were shared with the wider group to encourage open discussion and iterative reflection [36]. It is noteworthy that, initially, participants were instructed not to take images of their family members on the autism spectrum to reduce their worries about privacy. However, as trust deepened, some elected to share images of family members on the autism spectrum that prompted an ethics amendment. This evolution illustrates a core principle of CBPR: as trust grows, participants shape the research process and expand the scope of inquiry [30]. 

Session 4 focused on identifying patterns, concepts, and themes across 38 shared images and accompanying narratives. Prior to this session, the research team identified preliminary patterns to facilitate participant discussions. Session 4 also included a discussion on participants’ PhotoVoice journey and their preferred ways to share the findings with the broader community; this part of the discussion was audio-recorded and transcribed verbatim for key insights. An important step completed in Session 4 was consent about public sharing of participant-generated photographs and narratives. A tiered consent model was followed, where participants could choose whether their contributions for knowledge dissemination would be used anonymously, pseudonymously, or with attribution.

Data analysis followed the three-step PhotoVoice process of selecting, contextualizing, and codifying the images across the study sessions [27,37]. In Session 1, participants *selected* the topic areas for their photos. Next, during Sessions 2 and 3, participants *selected* photographs they felt were meaningful or significant to share with the group on selected topics. The *contextualization* occurred as participants described the thoughts, emotions, and experiences captured in their images, with others contributing questions, comments, and shared reflections. The SHOWeD technique guided these conversations by prompting participants to consider what they saw, what was happening, how it related to their lives, why the situation existed, and what could be done about it. Additionally, participants were encouraged to reflect on individual, family, community, system, and societal levels during these conversations, which led to rich analytic narratives for their photographs. A collaborative coding process followed. The research team members (FA and JS) reviewed all photographs and narratives to identify preliminary patterns, concepts, and themes. These were then presented to participants in Session 4 for discussion, ranking, and refinement. This participatory approach to codification aligned with CBPR principles of co-learning, community engagement, and co-creation of knowledge and ensured that the final themes reflected the collective analysis of both participants and researchers [30]. 

In terms of our approach to rigor and trustworthiness, we aimed for ethical engagement, shared ownership of the research process, participant-engaged analysis, and the potential to stimulate dialogue and policy influence [17,27,29]. Reflexivity was a central component. Throughout the study process, the first author kept a reflexive memo and took detailed notes to reflect on his thoughts, emotions, and decisions, which led to discussions with the research team for adjustments in the study procedures to account for blind spots and undue influence of researchers’ subjectivity. Further, a reflexive dialogue was embedded across all stages of this study, such as before and after each session, where the research team regularly reflected on how their social locations, experiences, and perspectives might shape data generation and interpretation. The team paid particular attention to adhering to the CBPR principles and, thus, employed strategies for participants’ active engagement in collective reflection and analysis, including opportunities to affirm, question, and refine emerging interpretations during group sessions. This participatory process helped ensure that the findings reflected participants’ priorities and meanings, rather than relying solely on researcher-driven interpretations.

## 3. Findings

Across 38 photographs and their narratives, seven themes were identified (Table 2). Each of these themes presented one or both domains that participants selected at this study’s onset, namely, Family/Community Supports and School/Education System. The domain of Family/Community Support was characterized most strongly by the theme of *Family Support and Child Needs*, followed by the *Caregiver Burden* theme. The School/Education domain was dominated by the theme of *School Support (or Its Missingness),* followed by the *Child Supports* theme. The themes of *Stigma and Discrimination*, *Journeys with Barriers*, and *Transitions and Uncertainty* emerged as crosscutting for the two domains. Here, we provide details on each theme using sixteen of the most representative photographs and accompanying narratives.

### 3.1. Theme 1. Family Support and Child Needs

**Figure 1:**
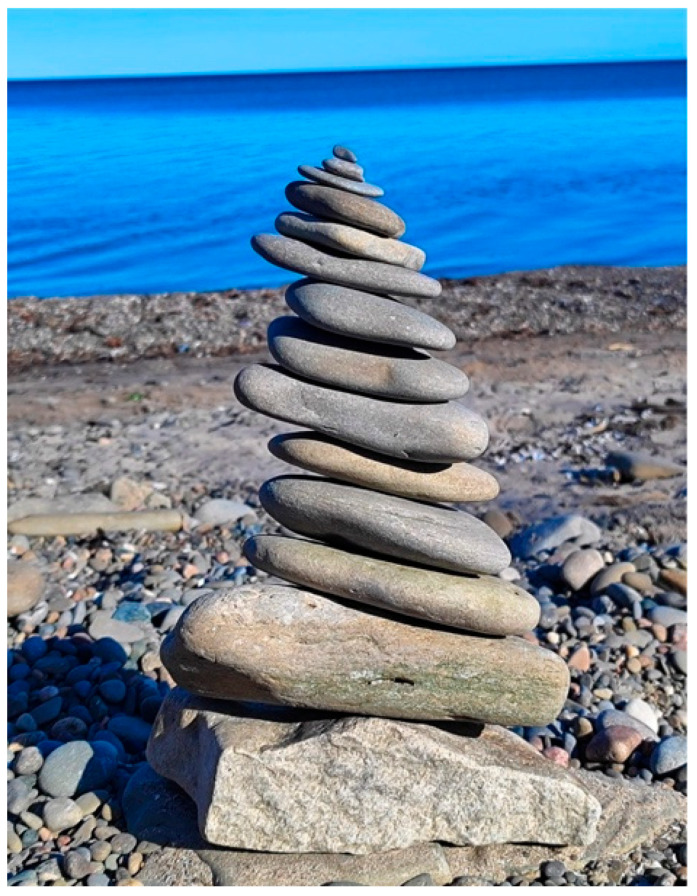
Foundation of care.

Many family caregivers in this study spoke about the dual realities of raising a child on the autism spectrum: the joy of close familial bonds and the challenges of meeting complex developmental needs. Families emerged not only as emotional anchors but also as primary systems of care, often filling gaps left by formal institutions. R.A., a caregiving mother, submitted a photo titled *Foundation of Care* (Figure 1), depicting a tower of stones stacked on a beach. She described the stones as representing the layered supports that are needed for a child to thrive, with family life forming the base:

“*Family relationships and a healthy home environment are key to a child’s development, especially for a child with autism. Family provides love, protection, and support*.”

—R.A.

At the same time, she likened caregiving to the delicate act of balancing stones, where each layer represents a responsibility that must be carefully managed to maintain stability.

“*Families can’t do it alone… At the government and health system levels, there is a need for financial, psychological, and social support*.”

—R.A.

Just as a tower can easily collapse if one stone is misplaced or missing, she emphasized that caregiving for a child with autism is precarious, vulnerable to strain, and heavily dependent on the presence of reliable external supports. Without these supports, the balance becomes fragile, and the weight borne by families alone can feel unsustainable. Her account highlighted how families often absorb the consequences when schools, therapists, or community services are limited, shouldering the costs at significant personal sacrifice.

**Figure 2:**
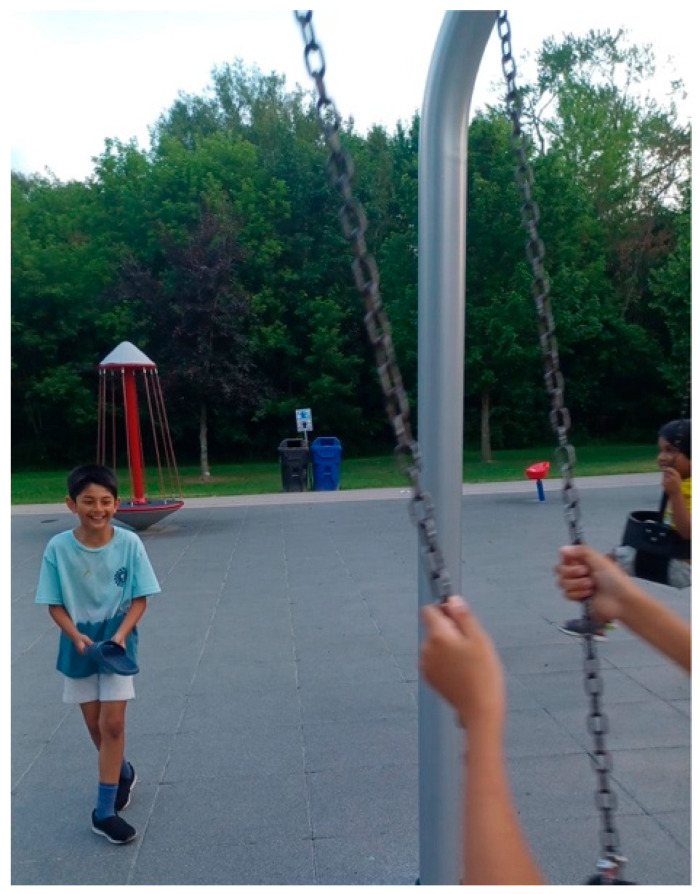
You are here.

Rubina, another mother, contributed a photo titled *You Are Here* (Figure 2), showing her son on a swing in a neighborhood park, with his younger sibling standing nearby. She explained that the image reflects the importance of unstructured play for her children’s learning and well-being:

“*My children are always asking to be entertained. ‘Take me to the park, Mama,’ or ‘Can we go to the park?’ They ask so many questions while they play. You can see different parts of their brains get activated. They require physical exertion to learn better and regulate their nervous system*.”

—Rubina

Her narrative highlighted the absence of adaptive and inclusive spaces within both schools and community settings that are essential for the child’s development. She explained that many environments fail to accommodate the sensory, physical, and social needs of children on the autism spectrum

“*There is a lack of attention at so many levels on creating physically inclusive and safe places for children on the spectrum. At least in the education system, more physical activities and play-based learning activities are required*.”

—Rubina

### 3.2. Theme 2. Physical and/or Emotional Burden on Family Caregivers

Caregivers described caregiving for a child on the autism spectrum as extending far beyond conventional parenting. They spoke about it as a physically demanding and emotionally exhausting role, often carried out with limited recognition or external support. The images highlighted how these responsibilities stretch across the lifespan, producing fatigue, fractured family relationships, and financial sacrifice.

**Figure 3:**
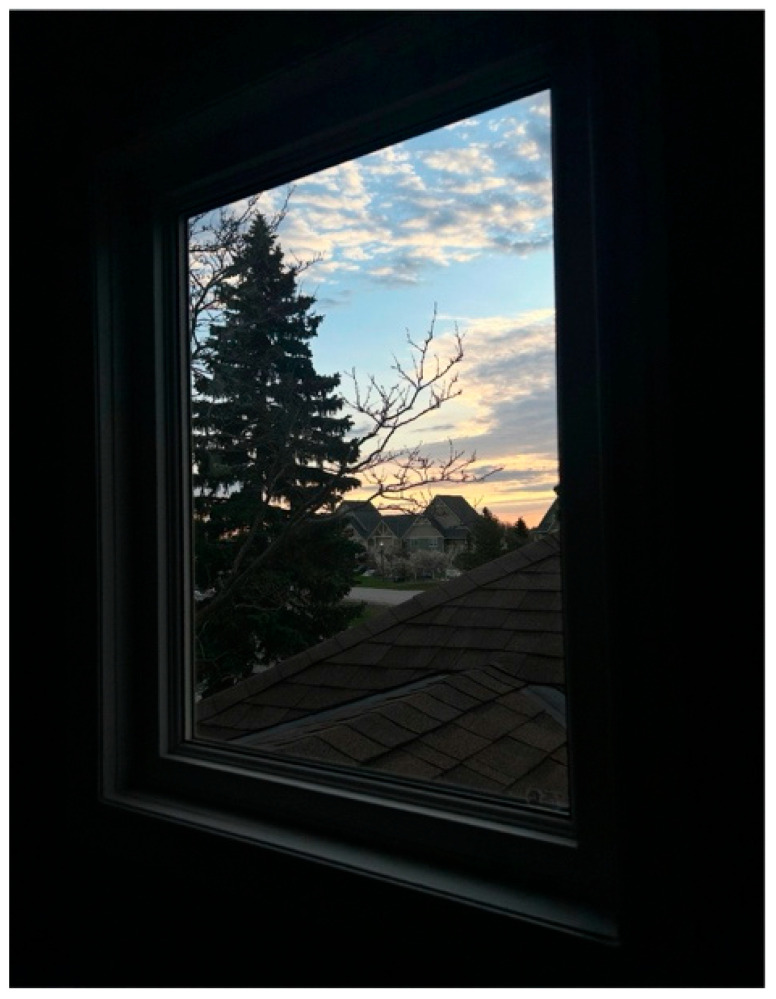
Sleepless nights, silent mornings.

Anonymous 1, a caregiving sister, shared a simple yet deep photo titled *Sleepless Nights, Silent Mornings* (Figure 3). The image juxtaposes a calm sunrise outside with the heavy stillness of the room inside. She explained that it represents the contrast between the quiet world outside and the exhausting realities of caregiving within the home. Her narrative focused on her brother’s irregular sleep patterns, which engaged the entire family:

“*He sometimes stays awake very late, wakes up early, and may scream or have meltdowns that wake everyone in the house… The lack of sleep affects our health, emotions, and ability to function, leading to burnout*.”

—Anonymous 1

She described how these “long, sleepless nights” left her “physically tired, emotionally drained, and still needing to keep going.” Alongside the physical exhaustion, she noted limited funding for in-home support and restricted access to behavioral specialists. In her view, caregiving was not adequately recognized, with families expected to continue meeting everyday obligations like work and school without appropriate assistance.

**Figure 4:**
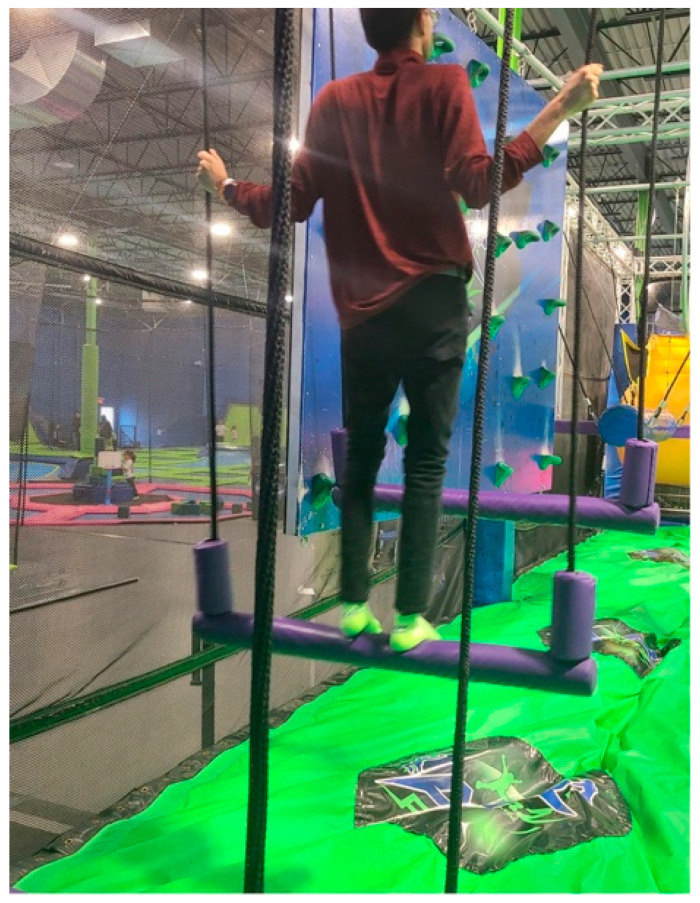
Parenting never pauses.

Hina, a caregiving mother, contributed an emotional photo titled *Parenting Never Pauses* (Figure 4). The image depicts her son navigating an obstacle course at a trampoline park with his father. She explained that despite his age, he requires constant supervisory support and cannot independently attend recreational or community spaces:

“*Our son never learned how to be completely independent… We are still doing most of the work and even creating recreational outings and planning their schedules. We have no break, we are always busy with work, and on the weekend, taking our son out to these places in the community, such as this trampoline park, so that he may exercise, enjoy, and develop his abilities*.”

—Hina

She added that her son did not have any respite worker at the time of the photo, making it “hard for us to do it on our own.” Her image emphasized the long-term nature of caregiving, which continues into adulthood when transition planning and adult services are unavailable or insufficient.

**Figure 5:**
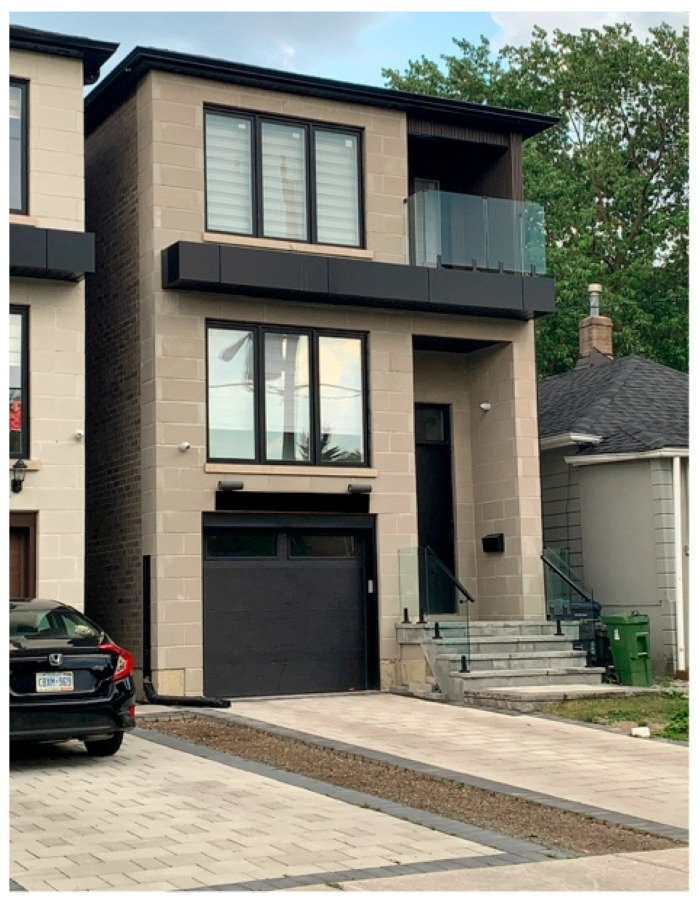
What we gave up and sacrificed.

Andrea, also a caregiving mother, shared a powerful photo titled *What We Gave Up and Sacrificed* (Figure 5). The picture shows two new houses near her own, which she and her family have not been able to renovate because of financial sacrifices made to cover therapy costs. For her, the newly built houses in the neighborhood serve as a daily reminder of what was given up:

“*My son looked at those new houses and said, ‘I don’t like our house. I like the new house. Can we move there?’ That moment broke my heart. He doesn’t see the hard choices we’ve had to make; he just sees what we don’t have*.”

—Andrea

Andrea explained that therapy costs left her family unable to invest in their home, and that navigating government funding programs and educational support felt confusing and inaccessible. Her photo conveyed both the emotional impact of her son’s innocent request and the material sacrifices made to provide him with necessary care. She further iterated that her child being on the spectrum is affecting her relationship and causing blame games:

“*And behind these choices, there is often pain between parents, too. Sometimes, one partner is blamed for the child’s diagnosis or for the financial struggles that follow*.”

—Andrea

### 3.3. Theme 3. School Support (Or Its Missingness)

Participants described schools as environments where children on the autism spectrum have often encountered misunderstanding, neglect, or insufficient support. Across images and narratives, caregivers highlighted deteriorating facilities, symbolic gestures of inclusion, and poorly aligned transition planning as examples of how schools failed to address children’s needs in meaningful ways.

**Figure 6:**
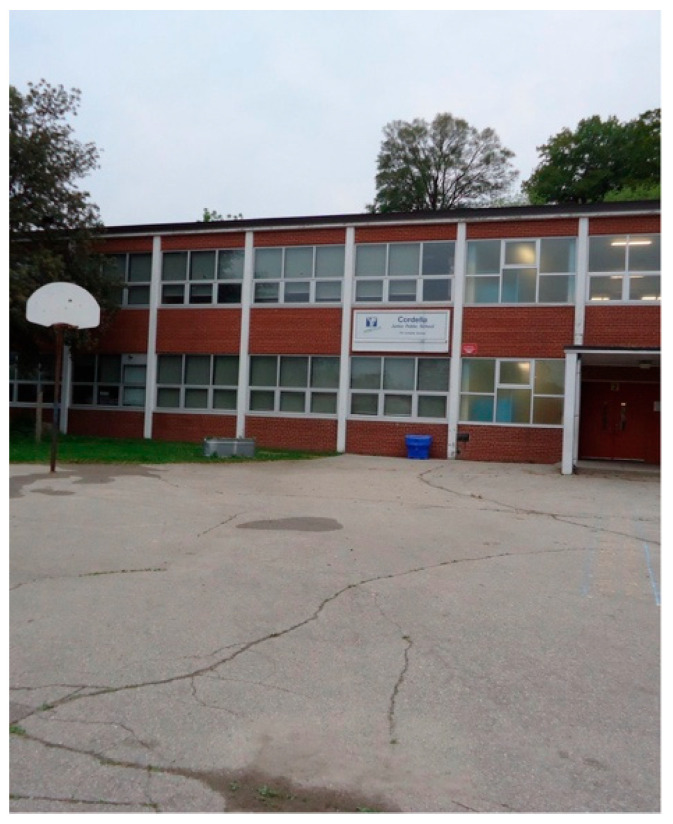
Neglected grounds, forgotten students.

Anonymous 2, a caregiving sibling, shared a photo titled *Neglected Grounds, Forgotten Students* (Figure 6). The image depicts a school ground marked by cracked pavement, sparse grass, and an old basketball court with missing nets. She explained that this was the common student line-up area, with only a small shelter for protection from rain. To her, the photograph conveyed “neglect” and the feeling of being overlooked:

“*You really have to live through it as a child to know what that feels like, what it’s like to spend every day in a space that’s falling apart. This connects to my life because my siblings spend most of their day at such a public school, and I want to know they’re somewhere that feels safe, clean, and cared for*.”

—Anonymous 2

She emphasized that all children “deserve the same quality of facilities and support as anyone else” and expressed a wish for students to feel proud of where they learn rather than “forgotten.”

**Figure 7:**
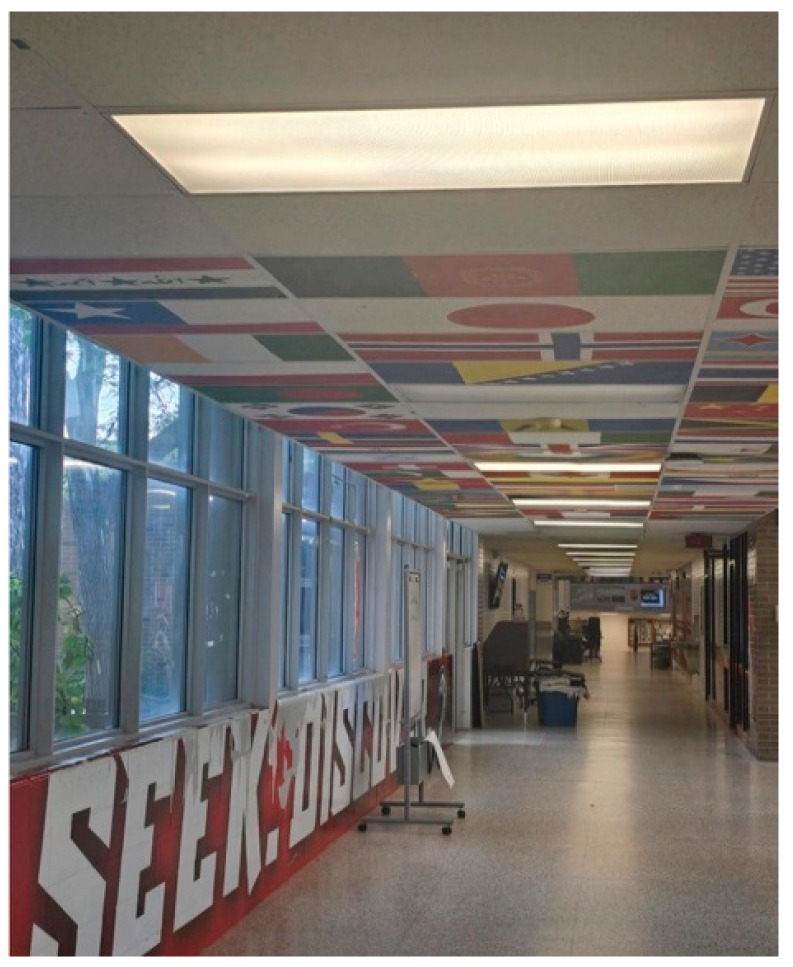
Beneath the ceilings of flags.

Shiraz, a caregiving mother, contributed a photo titled *Beneath the Ceilings of Flags* (Figure 7). The picture shows an empty school hallway, lined with international flags hanging from the ceiling, with indistinct windows at the far end revealing greenery outside. She described this image as representing her son’s experiences in school. While the flags suggested efforts toward inclusivity, she felt these attempts often failed to translate into meaningful support:

“*The flags represent some efforts by the education system to be inclusive for students with special needs, but the goals become blurry as children transition, such as from high school to adulthood*.”

—Shiraz

She elaborated that her son’s sensory needs were often overlooked and that schools tended to apply “one-size-fits-all” approaches. She noted that transition planning was especially difficult, as the school’s recommendation for work after graduation conflicted with her son’s demonstrated strengths in computers. Her family instead pursued a college pathway, believing it better aligned with his abilities.

### 3.4. Theme 4. Stigma and Discrimination

Stigma and discriminatory attitudes shaped their daily experiences as caregivers and siblings of children on the autism spectrum, participants mentioned. These accounts revealed how judgment, misunderstanding, and rigid expectations appeared across family interactions, community spaces, and educational environments.

**Figure 8:**
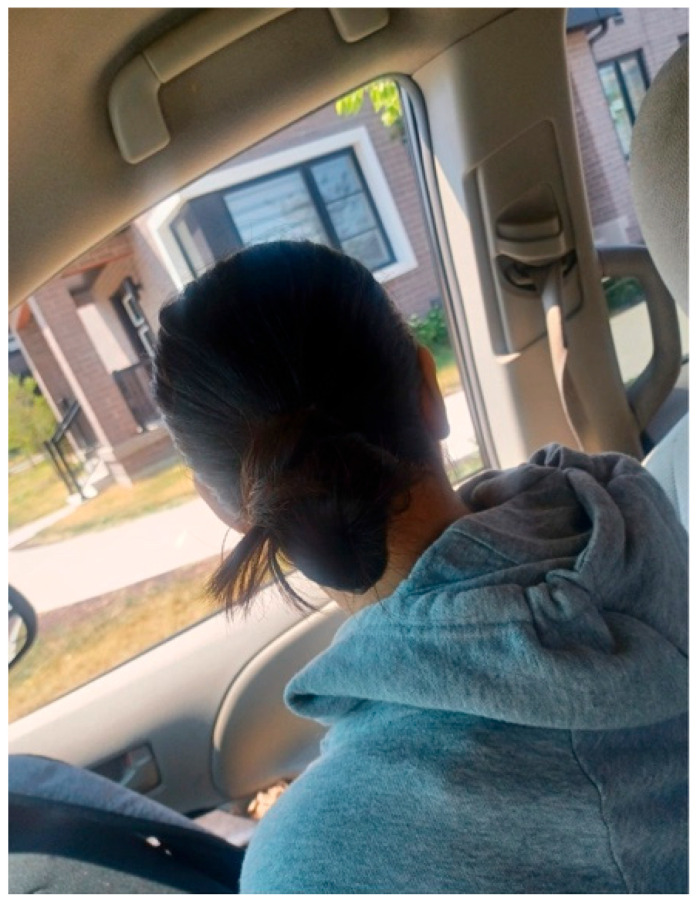
How do I look?

Rubina shared a photo titled *How Do I Look?* (Figure 8). The image shows her sitting in a car, with her daughter’s back facing the camera. She explained that the picture captured her ongoing worry about how others perceive her and her child:

“*It shows how I often feel worried about what others might see in us [caregivers and their children] that we can’t see in ourselves. It reflects my fear of how people look at me, and how I’m constantly thinking, ‘everyone is watching me.’*”

—Rubina

She further described how these fears extended beyond encounters with strangers to include her own family members:

“*I often feel judged, not just by strangers, but sometimes even by family… the fear of how we’re seen affects my confidence and how I move through the world*.”

—Rubina

**Figure 9:**
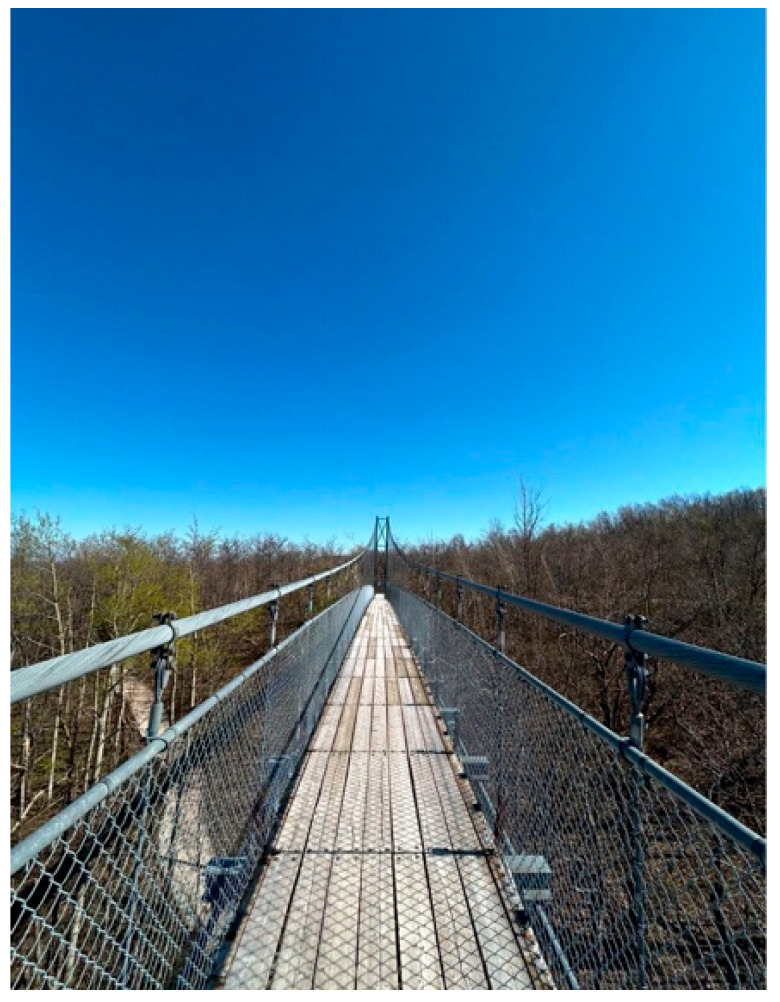
The standard path.

Anonymous 1 shared another photo called *The Standard Path* (Figure 9). The image depicts a narrow suspension bridge above bare trees, which she described as symbolizing the restrictive standards of the education system. She explained that schools often pushed children into rigid pathways that overlooked their strengths:

“*I’ve seen how the education system tends to push children with autism into narrow, standardized pathways. My brother is a clear example. He’s incredibly smart and especially skilled with technology*.”

—Anonymous 1

She emphasized that her brother’s talents were missed within these standardized approaches, which left little room for creativity or individualized learning. The photo conveyed her frustration at seeing his abilities unrecognized and her concern that the system’s structure limited his opportunities.

### 3.5. Theme 5. Overall Journey with Barriers

Participants expressed their caregiving journeys as marked by multiple barriers that limited the ability of their family members on the autism spectrum to experience inclusion, leading to disrupted routines, and added strain to family life. These barriers were often experienced as constant, recurring, and difficult to overcome, shaping both daily caregiving and longer-term planning.

**Figure 10:**
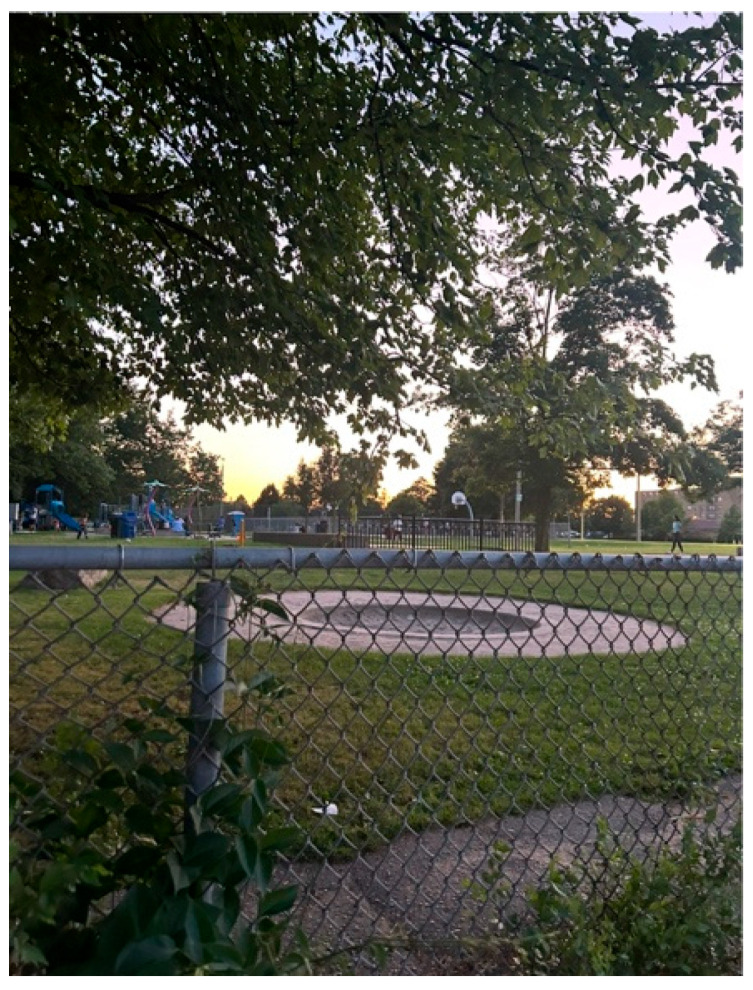
Barriers we face.

Anonymous 1 shared another photo titled *Barriers We Face* (Figure 10). The image shows a fence blocking the view of a park, behind which lies a sandy beach, a playground, and a basketball court. She explained that the fence symbolized the restrictions she and her brother face in living active and balanced lives:

“*The fence represents the barriers that caregivers of people living with autism face in living more active and balanced lives… It also stands for the communication barrier that comes with nonverbal autism*.”

—Anonymous 1

She described how her brother’s limited communication skills and sensitivity to loud or overwhelming environments often left him feeling separate from others and how her family’s routines had to be carefully planned to accommodate his needs.

**Figure 11:**
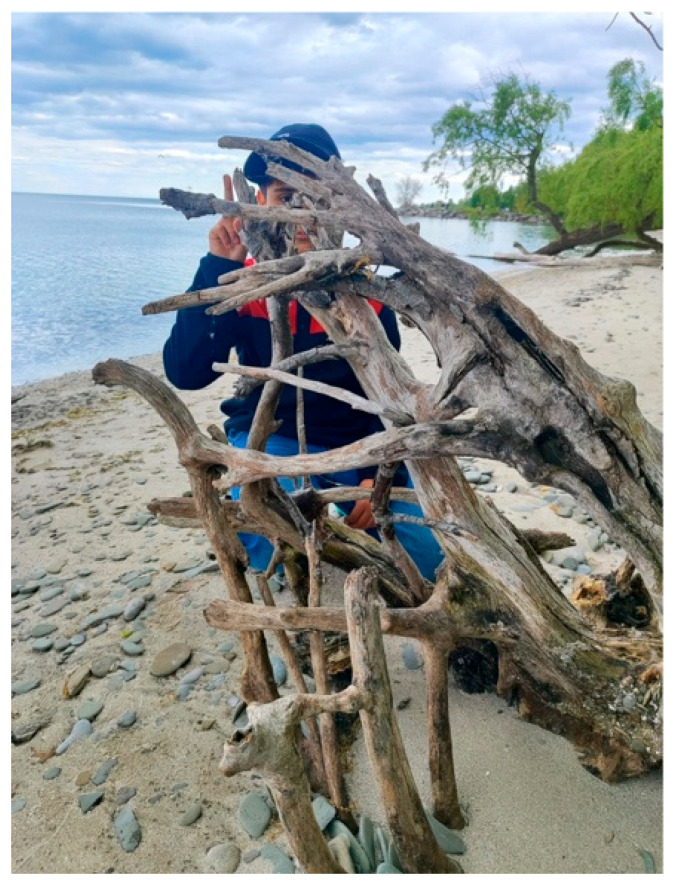
Under the branches/obstacles.

R.A. also presented a photo titled *Under the Branches/Obstacles* (Figure 11). The photo depicts a child looking out from behind an obstruction, which she described as symbolizing her son’s challenges in building connections with others:

“*It shows the many barriers that stop children from building strong relationships with their families, schools, and communities*.”

—R.A.

She reflected on the difficulty of understanding her son’s emotions and the unpredictability of triggers that could cause distress. This, she explained, often limited her ability to support him and deepened her concern that barriers in school and community settings further isolated him.

**Figure 12:**
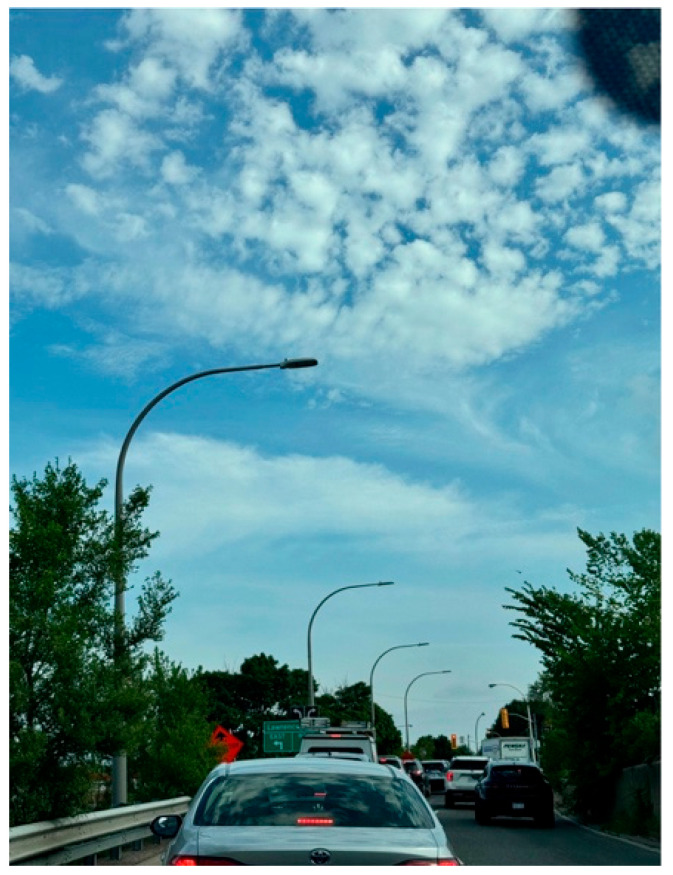
On my way home.

Zuhal, another caregiving mother, shared a photo titled *On My Way Home* (Figure 12). The image shows a road filled with construction signs and traffic congestion, taken during a drive home from school. For her, the photo represented the slow and frustrating process of navigating the education system:

“*To me, it reflects the slow and frustrating process experienced by individuals with autism and their families in schools and other life domains, like being ‘stuck in traffic’… Every school year, we start the process all over again from the beginning*.”

—Zuhal

She explained how frequent teacher changes and a lack of continuity in support disrupted her daughter’s school experience, causing stress and frustration for the entire family.

### 3.6. Theme 6. Transitions and Uncertainty

Whether across age, schooling, or cultural spaces, participants described these moments as filled with anxiety, hesitation, and a lack of clear guidance. For many, transitions felt like turning points where resources and pathways forward were unclear or unavailable.

**Figure 13:**
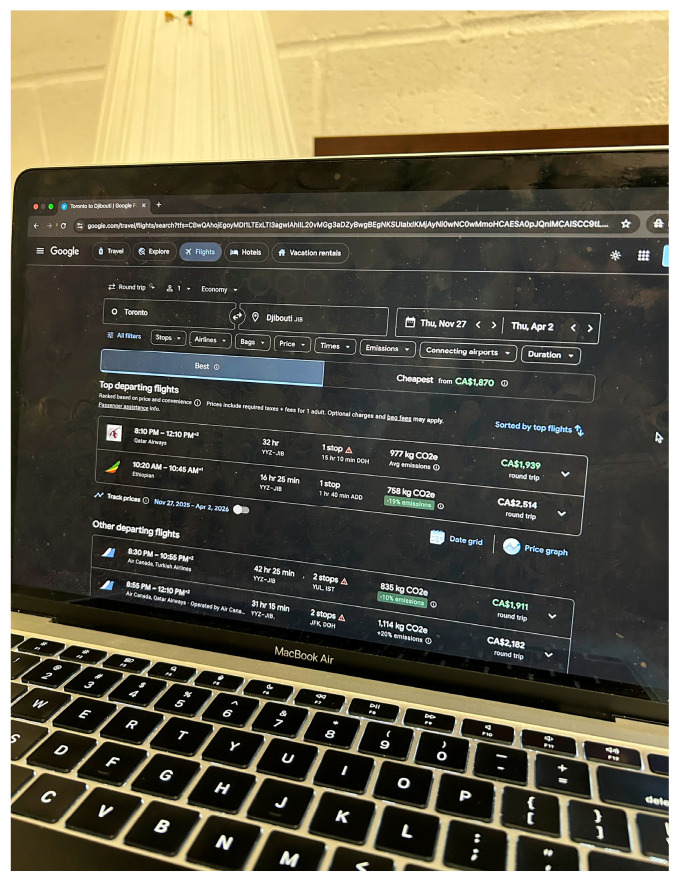
Intergenerational dilemma.

Sarah, an aunt caring for her nephew on the spectrum, shared a photo titled *Intergenerational Dilemma* (Figure 13). The photo depicts a computer screen showing a flight search from Toronto to Djibouti. Sarah explained that her mother believed a change of environment in their home country might “cure” her nephew’s autism and help him speak, while she herself felt he needed consistent schooling and routines. She described the conflict as an “intergenerational dilemma,” rooted in differing cultural beliefs and limited understanding of autism:

“*The situation exists because of varying cultural beliefs, limited awareness relating to children on the spectrum, and also the stigma around autism, where my mom thinks he can be ‘cured’ in a way*.”

—Sarah

**Figure 14:**
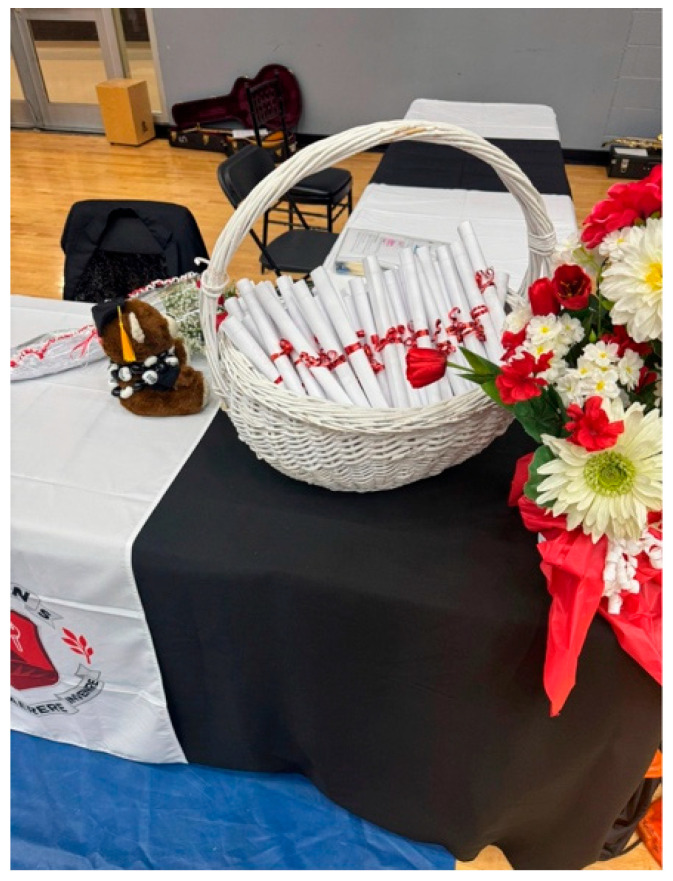
Graduation to the unknown??

Shiraz shared a photo titled *Graduation to the Unknown??* (Figure 14). The image shows a teddy bear dressed in graduation attire, sitting beside flowers and a basket of rolled-up diplomas. For Shiraz, the teddy bear symbolized her son on the autism spectrum as he approached graduation: proud yet unprepared and moving into an uncertain future. She described her feelings as both joyful and worried:

“*Is society ready for him? Can he speak up for himself? How much does he understand about what’s ahead*?”

—Shiraz

Her photo and narrative reflected the bittersweet emotions of celebrating her son’s achievement while fearing the lack of clear support for life after high school.

### 3.7. Theme 7. Two Sides of a Coin: Isolation and Strength, Loneliness and Hope

The final theme reflected a powerful duality in caregivers’ lives: the weight of loneliness and isolation alongside moments of hope, resilience, and family strength. Participants described living in these tensions and daily navigating systems that felt unwelcome while drawing endurance from their closest relationships.

**Figure 15:**
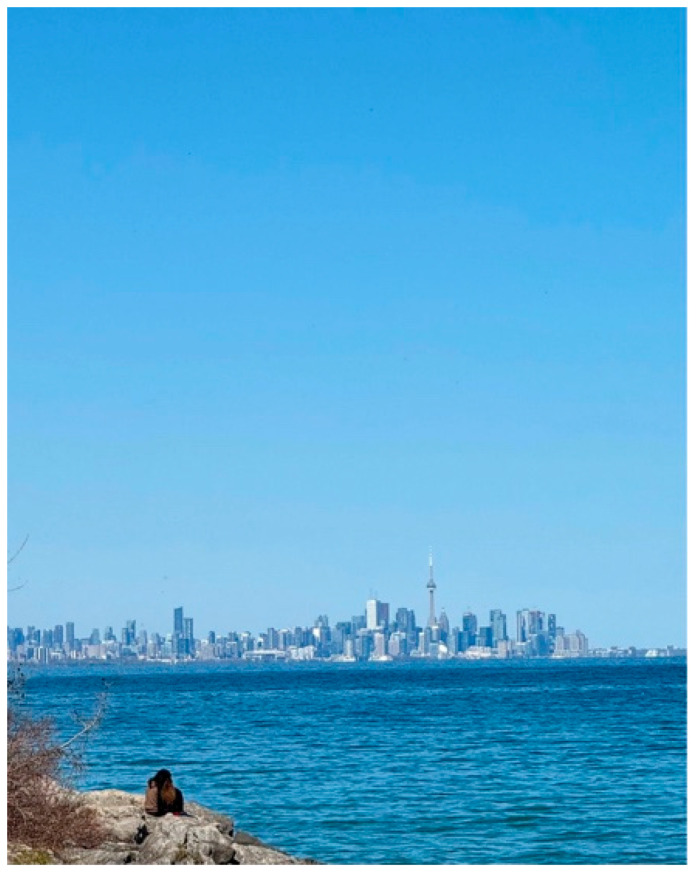
Isolation and navigation in a new city.

Zuhal shared a photo titled *Isolation and Navigation in a New City* (Figure 15). The image shows a couple seated on a rocky shoreline, looking toward the distant Toronto skyline. She explained that the city symbolized the support and belonging that always seemed visible yet far away. She described the photo as representing both the strength of parents raising a child on the autism spectrum and the isolation that comes with navigating unfamiliar systems as newcomers:

“*This photo represents how parents of neurodivergent kids feel isolated while navigating systems and resources, due to a lack of understanding and knowledge about autism. As parents, we often feel alone and like outsiders. We are new to this country and don’t understand many of the systems. That makes us feel tired, confused, and emotionally distant*.”

—Zuhal

For her, immigration added further layers to the caregiving journey, including language barriers, stigma, and the absence of extended family support. Yet she also described the strength that comes from “relying on each other” as a couple, highlighting the coexistence of struggle and resilience.

**Figure 16:**
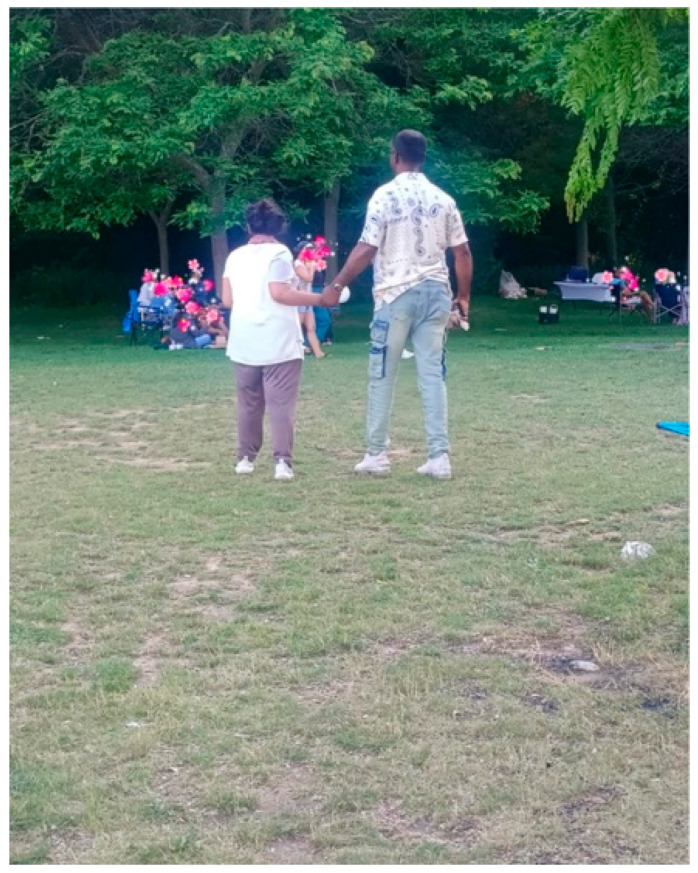
Fathers can also do it, the Caregiving!

Sonam, a mother caring for her daughter, contributed a photo called *Fathers Can Also Do It, the Caregiving!* (Figure 16). The image depicts a father and daughter walking hand in hand. For Sonam, it symbolized both joy and trust, as well as the strength of shared parenting:

“*As a mom of a daughter with autism, I am so proud of my husband, who works six days a week and still finds time on Sundays for caregiving*.”

—Sonam

Participant empowerment was another key objective of this participatory research, and it was vividly demonstrated throughout the Photovoice process. At the outset, several participants expressed apprehension about engaging in photography or sharing their personal stories in a group setting. As one participant reflected:

“*Initially for me, I thought it was just one session…and I didn’t think I could come up with even one picture. So, it was very difficult. But after the second session, it was easier and really hearing everyone’s story made me feel like… you are one of [us] and it normalizes the situation*.”

—Shiraz

As the sessions progressed, the group evolved into a supportive, reflexive space where participants felt increasingly comfortable, confident, and connected. This empowerment was evident not only in their storytelling but also in their growing sense of community. For example, participants described how sharing and listening transformed isolation into solidarity:

“*I came here to meet people with the same condition as mine… it was a great reflective journey for me because I look back at my own journey and what else I can do for my child. I learned from everyone else*.”

—Zuhal

“*Here, I didn’t really talk to many people about caregiving before. It really opened up a new perspective… Caregiving for autism isn’t just one way, there are so many perspectives*.”

—Anonymous 1

Participants in this study also advocated assisting in disseminating the results within school boards and engaging university students, recognizing them as the next generation of policymakers. For example, participant caregivers explained,

“*Maybe having a gallery of some of the works… in school board transition evenings… someone to talk about what came out of the families*.”

—Shiraz

“*It’s important to present it at universities… they are the next policymakers and change makers*.”

—Anonymous 2

The group’s collective dialogue cultivated a sense of mutual learning and belonging. Participants described the sessions as *“a family,” “a unit,” and “a therapy group,”* noting that *“you get exposed to something like this and it wakes you up… you think differently”*—Rubina. Through these exchanges, participants felt empowered beyond the research context as they began envisioning their advocacy roles and collective action after the research. For instance, participants proposed developing policy briefs, community exhibits, and presentations for schools and government bodies to “raise awareness” and “bring solid change.” Empowerment thus manifested at multiple levels: emotional, social, and political. Participants not only generated data but also built lasting relationships, exchanged coping strategies, and committed to staying connected through a WhatsApp group and future dissemination initiatives. As one participant summarized,

“*Everyone here is like a big family… We can share, feel better, and push ourselves. The government and education system must pay more attention, but we are already doing something together*.”

—R.A.

## 4. Discussion

**Figure 17:**
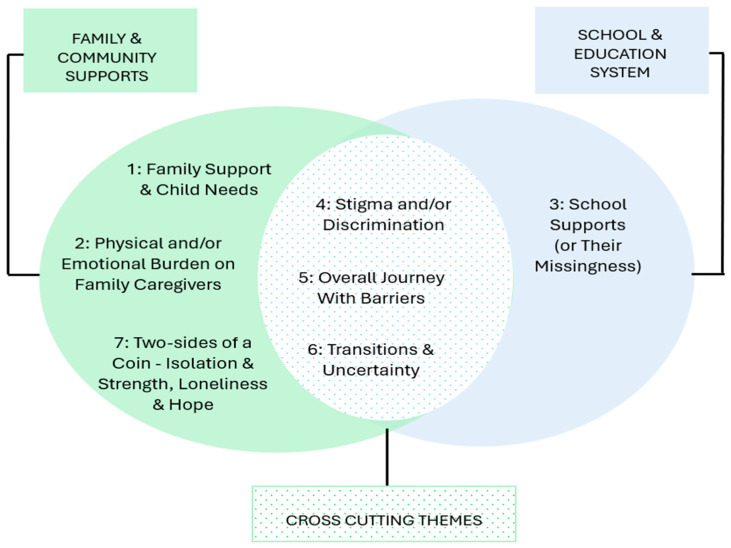
Domains and cross-cutting themes emerging from the PhotoVoice study.

This Photovoice study explained how immigrant and racialized caregivers of children on the autism spectrum in Canada experience and make sense of caregiving within the social structures that both sustain and constrain them. Guided by the socioecological framework and our theoretical lenses, the findings reveal that family, community, and institutional contexts are deeply intertwined. At the micro level, caregiving unfolds in intimate acts of love and adaptation; at the meso and macro levels, it is shaped by educational inequities, health-system barriers, and immigration structures. Figure 17 captures these intersecting layers, illustrating how stigma, systemic barriers, and transitions link family and institutional domains.

### 4.1. Family Support and Child Needs

Families emerged as the foundation of care for both emotional and instrumental support for the child on the spectrum. Their instrumental support included activities left unfulfilled by formal institutional support. Participants described providing a constant support structure, advocacy, and learning opportunities for their family members on the autism spectrum, yet doing so with limited access to culturally or financially appropriate services. Their accounts reveal that family is not a private sphere detached from the formal systems of care, but a site where policy failures become personal strain and exhaustion. These findings echo the results of studies by Casale et al. [11] and Khanlou et al. [14], who documented how immigrant caregivers’ shoulder structural gaps through unpaid labor and emotional endurance. In our study, the imagery of balancing stones and playground swings in the participants’ photos symbolizes this delicate equilibrium between love and exhaustion. Policy responses must, therefore, move beyond clinical interventions for the child on the spectrum to strengthen family-centered community support, such as culturally grounded respite programs and family mentoring networks that recognize caregiving as a collective social infrastructure rather than an individualized obligation. Further research on co-designing family-centered programs is much needed and emphasized in Canada’s National Autism Strategy of 2024 [38].

### 4.2. Caregiver Burden

Participants portrayed caregiving as an all-consuming role that extends well beyond ordinary parenting. Sleepless nights, financial sacrifices, and marital tension underscored the invisible costs of sustaining the development of their family members on the autism spectrum. Within the socioecological framework, these pressures reside at the intersection of the micro- (home), exo- (policy), and macro- (social values) systems, where inequitable access to resources magnifies fatigue and burnout. Consistent with studies by Woodgate et al. [8] and Shafi et al. [13], the absence of affordable therapies and respite support deepens families’ precarity. Yet, caregivers also redefined hardship as purposeful to resist against marginalization, given their immigrant and/or racialized status. Recognizing this duality demands systemic reform: expanding publicly funded counselling coverage for caregivers’ stress, embedding flexible funding for home-based respite, and ensuring that service eligibility and access are not influenced by the caregivers’ immigration or minority status. Supporting caregivers is not a charity; it is an investment in community resilience and public health equity.

### 4.3. School Support (Or Its Missingness)

Caregivers’ photographs of deteriorating school grounds and empty hallways lined with multicultural flags reveal a painful contradiction: schools that celebrate diversity symbolically while failing to meet the concrete needs of neurodivergent students. Participants described how under-resourced schools, low teacher expectations, and fragmented transition planning communicated the invisibility of their family members on the spectrum. These findings align with research by Rivard et al. and Fong et al. [39,40], who found that immigrant families frequently experience diminished trust in education systems that prioritize compliance over inclusion. Others have reported discrimination experienced by immigrant families in the Canadian school system [41]. For racialized children on the spectrum, exclusion also operates subtly through environments that are physically unsafe and pedagogically inflexible. To address this, education policy must go beyond token multiculturalism. The government ministries of education and children’s well-being should work collaboratively so that schools receive enhanced funding for accessibility, such as in the Canadian province of Saskatchewan [42]. Likewise, there is a strong need to co-develop transition plans with families that emphasize students’ strengths rather than their perceived deficits. Likewise, the teachers’ training colleges ought to integrate training on cultural humility and neurodiversity into teacher preparation. 

These findings align closely with the social model of disability, which conceptualizes disability as arising from social and environmental barriers rather than from individual impairment [20]. Participants’ accounts indicate that exclusion was produced not by their children’s abilities, but by rigid school structures, inflexible pedagogical approaches, and limited or poorly coordinated transition planning. From a neurodiversity perspective, caregivers did not frame their family members on the autism spectrum as deficient or lacking; instead, they emphasized their strengths, interests, and capacities, which were frequently overlooked within systems oriented toward standardization and normative performance benchmarks [21,22,23]. The disjuncture between children’s capacities and institutional expectations highlights how educational environments continue to privilege normative developmental trajectories. As a result, neurodivergent learners are rendered marginal or invisible in practice, despite policy-level commitments to inclusion. Together, these findings show the persistence of structural barriers within educational systems and reinforce the need to move beyond symbolic inclusion toward pedagogical and organizational practices that meaningfully accommodate neurodiversity.

### 4.4. Cross-Cutting Challenges: Stigma, Barriers, and Transitions

Stigma and systemic barriers are threaded through every aspect of caregiving. Participants spoke of feeling judged by relatives and dismissed by service providers, illustrating how racism, ableism, and cultural misunderstanding intersect to limit both belonging and access. Their “overall journey with barriers” was less a series of isolated obstacles than an enduring condition of navigating misaligned systems. These findings echo what Khanlou et al. [14] describe as the “double jeopardy” of racialized disability, where caregivers contend simultaneously with prejudice and bureaucratic fragmentation. Other Canadian scholars also report on the transition challenges among immigrant or racialized families caring for children living with autism or other disabilities [14,41]. The neoliberal orientation of Canada’s autism policy landscape, as noted by Shepherd and Waddell [43], compounds this by privatizing responsibility for care. In our study, participants’ photos of fences, construction zones, and uncertain pathways capture this climate of endurance within constraint. Transitions, whether developmental, educational, or cultural, were described as moments of acute vulnerability. The anxiety over post-graduation futures or intergenerational conflict around autism beliefs underscores how systemic coordination fails precisely when stability is most needed. Addressing these intertwined challenges requires coordinated intersectoral policy: culturally informed stigma-reduction initiatives, simplified funding systems, and early, family-driven transition planning that links schools, health agencies, and community organizations. Several of these are identified as essential in Canada’s National Autism Strategy of 2024 [38], though the current economic downturn may push this to the back burner.

Caregivers’ narratives in this study directly challenge deficit-based interpretations of autism. Across themes, participants emphasized adaptation, creativity, and relational care, highlighting that experiences of distress emerged primarily when social systems failed to respond to neurodivergent needs. Rather than locating challenges within the child on the autism spectrum, caregivers’ accounts pointed to systemic constraints that limited flexibility, responsiveness, and meaningful inclusion. In this regard, the findings support a shift from framing autism-related challenges as individual or family-level problems toward understanding them as outcomes of policy design, fragmented service delivery, and prevailing social attitudes. Such a reframing is central to both social model and neurodiversity-informed approaches and shows the importance of structural and policy-level interventions that move beyond individualized accommodations toward systemic change.

### 4.5. Isolation and Strength (Dualities of Caregiving)

Perhaps the most profound theme was the coexistence of loneliness and resilience. Caregivers described feeling unseen by institutions yet deeply connected to one another through shared experience. Their reflections reframed caregiving as both burden and solidarity, and a site of emotional survival and quiet activism. The study process itself, through PhotoVoice, became a vehicle of empowerment: participants formed peer networks, exchanged coping strategies, and envisioned advocacy beyond research, including dissemination through school boards and universities. Such empowerment affirms the transformative potential of participatory methods. As Catalani and Minkler [29] argue, CBPR generates not only data but also agency. The participants’ commitment to continue collaborating through a WhatsApp group and community exhibits exemplifies this shift from research subjects to knowledge partners.

### 4.6. Limitations

Given the qualitative and participatory design of the reported study, transferability of the findings warrants some caution. We engaged with ten immigrant and racialized family caregivers who were all women—seven mothers, two sisters, and one aunt—and residents of a metropolitan area in Ontario. Their experiences may not reflect all family caregivers and those residing in other regions, including Canada, where publicly funded health and social care programs are specific to each province and territory. In terms of gender representation, all participants who enrolled in the PhotoVoice study were women despite a broad recruitment strategy. This is a possible consequence that women disproportionately assume primary caregiving responsibilities for children and family members living with disabilities. Nevertheless, future research designs should intentionally engage caregivers of all genders to explore how caregiving roles, burdens, and support needs may vary across genders. In terms of analysis, given the small number of participants and to avoid the risk of essentialism, we could not analyze variations by the participants’ migration histories, family roles, and cultural contexts that were sometimes reflected in individual narratives. Future scholarly work would benefit from examining both intra-group and inter-group differences and similarities among diverse immigrant and racialized caregivers by using study designs and sample sizes that support meaningful comparative analyses across ethnic, cultural, and social contexts. At the same time, the contextually grounded issues of caregiving burdens, school-based inequities, and systemic barriers are likely to resonate in other immigrant-receiving settings, highlighting the value of participatory approaches in identifying and addressing sensitive and underexplored issues.

## 5. Conclusions

In conclusion, family caregiving to children and youth on the spectrum emerges in our study as both a profound act of love and a site of structural injustice. Furthermore, immigrant and racialized caregivers are not passive recipients of services but active agents navigating inequitable systems with creativity and courage. Through the socioecological and critical social science lens, their personal struggles reveal several policy failures and systemic inequities that can no longer be framed as private matters. Strengthening equity in autism policy demands centering these caregivers’ voices, redistributing support, and embedding cultural responsiveness into every level of care.

## Figures and Tables

**Table 1:** Sociodemographic characteristics and number of participants.

Ethnic Descent	Age	Employment Status
East African—3	18–25 yrs—1	Full-time—3
South Asian—3	26–35 yrs—3	Part-time/self-employed—2
Eastern European—2	36–50 yrs—3	Unemployed—5
Middle Eastern—1	51–60 yrs—3	
Southeast Asian—1		
**Relationship with Child**	**Born in**	**Social Support □**
Mother—7	Canada—3	Poor or fair—2
Aunt or sibling—3	Other—7	Good—5
		Very good or excellent—3

□ Scale of 1–5: poor, fair, good, very good, excellent.

**Table 2:** Seven themes across thirty-eight photographs and narratives.

1. Family Support and Child Needs	2. Physical and/or Emotional Burden on Family Caregivers	3. School Support (or Its Missingness)	4. Stigma and Discrimination
Families provide both love and essential care while struggling to fill the gaps left by inadequate formal supports.	Caregiving places heavy physical, emotional, and financial strain on families, often leaving mothers exhausted and unsupported.	Lack of meaningful school support leaves caregivers and children feeling overlooked and excluded within the education system.	Captures how families experience stigma and discrimination, which shapes their daily struggles and sense of belonging.
**5. Overall Journey with Barriers**	**6. Transitions and Uncertainty**	**7. Two Sides of a Coin:** **Isolation and Strength,** **Loneliness and Hope**	
Ongoing challenges families face, showing how systemic barriers make caregiving for children with autism especially difficult.	Families face stress and confusion during major life transitions, with little guidance or support to ease the process.	The dual reality of caregiving, where feelings of isolation and loneliness exist alongside love, resilience, and hope within families.	

## Data Availability

The photos and narratives can be made available upon reasonable request to the corresponding author.
